# Trauma Stabilisation as a Sole Treatment Intervention for Post-Traumatic Stress Disorder in Southeast Asia

**DOI:** 10.1007/s11126-018-9598-z

**Published:** 2018-09-28

**Authors:** Cordula Eichfeld, Derek Farrell, Marcel Mattheß, Peter Bumke, Ute Sodemann, Nil Ean, Bunna Phoeun, Yulia Direzkia, Filino Firmansyah, Nathanael E. J. Sumampouw, Helga Mattheß

**Affiliations:** 1 Duisburg, Germany; 2 University of Worcester, Worcester, UK; 3 Trauma Aid Germany, Duisburg, Germany; 4 Berlin, Germany; 5 Royal University of Phnom Penh, Phnom Penh, Cambodia; 6 EMDR Cambodia Association, Phnom Penh, Cambodia; 7 Banda Aceh, Indonesia; 8 EMDR Indonesia Association, Jakarta, Indonesia; 9 Universitas Indonesia, Depok, Indonesia

**Keywords:** Post-traumatic stress disorder, Trauma stabilisation, Cultural sensitivity, Southeast Asia, Therapeutic relationship, Trauma capacity building

## Abstract

Southeast Asia contains high numbers of traumatised populations arising from either natural disasters or interpersonal violence. Consequently, the need for empirically based trauma treatments, compromised by insufficiency in appropriately trained clinicians and mental health workers, makes the situation more challenging in addressing traumatic sequelae in local populations. In response, the humanitarian/ trauma capacity building organisation, Trauma Aid Germany, trained 37 therapists in psycho-traumatology, based on EMDR Therapy, which included trauma stabilisation techniques. This research analyses the impact of Trauma Stabilisation as a sole treatment intervention for Post-Traumatic Stress Disorder (PTSD) in adults. Each client was screened for PTSD utilising the Harvard Trauma Questionnaire - pre- and post-treatment. Analysis of the data considered only those interventions focussed on trauma stabilisation, including psychoeducation. Participants receiving trauma confrontation interventions were excluded from the data. Trauma stabilisation - as a sole treatment intervention, was highly effective in alleviating PTSD diagnoses. Results demonstrate PTSD symptoms were reduced in both clinical and sub-clinical trauma groups. The data set suggests trauma stabilisation, as a sole treatment intervention, was safe, effective, efficient and sufficient treatment intervention for PTSD. Furthermore, trauma stabilisation interventions have the advantage of being safe, flexible, and adaptable to the cultural and spiritual context in which they were are applied. The research findings also have implications regarding teaching and learning and the potential utilisation of paraprofessionals, and other allied health professionals in addressing the global burden of psychological trauma.

## Introduction

The Centre for Research on Epidemiology of Disasters (CRED) [1] reports, Asia as the continent most affected by natural disasters (44.4%), has the most disaster victims (69.5%) and suffers the most damage (64.4% of worldwide natural disasters reported costs). An example of this relates to the Indian Ocean Tsunami in Southeast Asia in December 2004. This natural disaster was responsible for the death of 225,841 people making it the sixth deadliest natural disaster in the world [2]. Post tsunami a survey by Souza et al. [3] carried out in Aceh Province, Indonesia, determined that 83.6% of survivors demonstrated signs of emotional distress and 77.1% depressive symptoms. Levels of emotional distress increased the more individuals were exposed to tsunami-related deaths among household members. Further post-tsunami studies highlight a prevalence of Posttraumatic Stress Disorder (PTSD) - 8.6 to 57.3% among Asian survivors of natural disasters. However, this was highly dependent upon methodologies and diagnostic instruments used [2, 4].

Apart from natural disasters Southeast Asian populations suffers from various kinds of interpersonal violence. The World Report on Violence and Health [5] defines violence as “The intentional use of physical force or power, threatened or actual, against oneself, another person, or against a group or community, that either results in or has a high likelihood of resulting in injury, death, psychological harm, mal-development or deprivation” (p. 5). It further distinguishes self-directed (inflicted upon person him−/herself), interpersonal (inflicted by another individual/small group of individuals) and collective violence (inflicted by larger groups such as organized political groups and terrorist organisation). Interpersonal violence is divided into family and intimate partner violence (usually taking place in the home, also called domestic violence) and community violence (violence between unrelated individuals, generally taking place outside the home) [5].

An example of violence being perpetrated on an industrial scale relates to the acts of genocide, perpetrated by the Khmer Rouge regime in Cambodia between 1975 and 1979. Kiernan [6] estimates that this brutal regime was responsible for the deaths of between 1.67 and 1.87 million people – some 20% of the entire Cambodian population. The Khmer Rouge were responsible for mass executions, persecution and perpetrating a regime of terror. As van Schaack et al. [7] purports, the impact on mental health continues to be in evidence with regard to PTSD, Depression and other severe mental health problems.

### Availability of Therapists and Mental Health Services

At the same time, the education of the therapists in the region is quite poor and Mental Health services are very rare. In Cambodia there are 63 mental health outpatient facilities resulting in a rate of 0.42 per 100.000 people [8] and in Thailand 93 mental health outpatient facilities resulting in a rate of 0.14 per 100.000 people [9]. For comparison, in Germany there are 24.881 mental health outpatient facilities resulting in a rate of 30.32 per 100.000 people [10]. For Indonesia there is no information available [11].

### Trauma Aid and the Mekong I Project

Trauma Aid (HAP Germany) is a Humanitarian Organisation, which aims to establish Trauma Capacity Building/ Psychotherapeutic services in crisis areas like Southeast Asia [12]. The primary objective of the organisation is to train local health workers and non-governmental organisations (NGOs), in psycho-traumatology, trauma interventions, and trauma self-care based upon empirical research and international treatment guidelines [13–15]. Within the Trauma Aid Germany portfolio was the establishment of Mekong I [2010–2014] – a Trauma Capacity Building Project for Thailand, Cambodia and Indonesia - Mekong I was funded by the German Federal Ministry for Economic Cooperation and Development – also in co-operation with Terre des Hommes (Germany). One of the primary objectives is to increase the mental health – trauma capacity focussed on evidence-based treatment interventions. This involved teaching and learning, skills training, diagnostic screening, and trauma treatment interventions which included psychoeducation, stabilisation techniques and trauma processing (confrontation) mainly centred upon Eye Movement Desensitization and Reprocessing (EMDR) Therapy and empirically validated psychological treatment intervention for PTSD. There were 37 therapists trained during Mekong I of which their professional background was that they are either psychologists or psychiatrists.

### Rationale for the Research

Herman [16] presented a model of trauma treatment that involves three critical phases:

- Phase 1: Safety and Stabilisation
- Phase 2: Remembering and Mourning
- Phase 3: Reconnection

This three-phases approach is the recommended approach for treating PTSD, especially complex cases – as recommended by the current ISTSS guidelines for the treatment of complex PTSD [17]. Phase 1 – Safety & Stabilisation, focuses on the insurance of the individual’s safety, reducing symptoms and increasing emotional, social and psychological competences. It also includes psychoeducation providing explanations to account for client’s symptoms and experiences, giving the client hope for resolution and providing, when appropriate, a sense of normalisation when necessary. Phase 2 – Remembering & Mourning is ostensibly a ‘Trauma Confrontation’ phase, focuses on the processing of unresolved aspects of the individual’s memory of the adverse life (traumatic) experience. WHO [18] empirically supports Trauma-Focused Cognitive Behavior Therapy (TF-CBT) and EMDR therapy as efficacious treatments for PTSD. Phase 3 – Re-connection, focuses on the consolidation of change and moving forward [16, 19, 20]. There is evidence that the therapeutic alliance and negative mood regulation achieved in Phase 1 predicts the success in reducing PTSD in Phase 2 emphasising the value of establishing a strong therapeutic relationship and emotion regulation skills before exposure work, especially among chronic PTSD populations [21–23]. This fits with Asay and Lambert [24], Lambert [25] who indicate that the therapeutic relationship accounts for 30% of client improvement (while specific techniques account for only 15%).

#### Stabilisation

The Mekong I project wanted to consider the impact of trauma stabilisation as a sole treatment intervention in PTSD. Research supports the activation of the patients’ strength and resources as an important change mechanism in psychotherapy [26–28]. Especially with complex cases following childhood abuse, trauma-specific stabilisation has a prominent role. It includes techniques of attention focussing, the focussed use of imaginative distancing techniques and the use of resource activating techniques [20, 29]. Techniques of attention refocusing direct the attention away from the traumatic internal experience toward stimuli of the external (neutral or positive) reality resulting in reduced distress. Imaginative distance techniques aim at reducing the traumatic affect and enhancing the feeling of control and safety. Imagination techniques well established are the “container” technique and the “safe place” exercise [20, 29, 30]. Resource activation is based on the principle that positive emotions can reduce the impact of negative emotions [31]. Resources can be activated through imagination evoking positive memories, personal successes, positive relationships and role models [29, 32, 33].

The content of trauma stabilisation taught in the Mekong I training is included in the treatment manual of Wöller and H. Mattheß [34] for Resource-oriented trauma therapy combined with EMDR resource installation - ROTATE. ROTATE itself is not a form of Trauma Stabilisation – but instead a manualisation of a multitude of different trauma stabilisation techniques, strategies and interventions. The approach aims at strengthening resilience and coping capacities by activating positive personal resources within a secure therapeutic relationship. A variety of imaginative resource-activating methods are included and based on a framework informed by affective neuroscience, resilience research, and attachment theory. Despite containing EMDR therapy elements and techniques, trauma stabilisation does not involve trauma confrontation or working with trauma memories. A RCT has shown that symptoms of PTSD and other co-morbid trauma-related symptoms (like depression and anxiety) can be effectively reduced using trauma stabilisation interventions as outlined in the ROTATE manual and subsequent research study [35, 36].

There are distinct advantages to trauma stabilisation as a specific intervention - it can be safely applied, is language-independent and flexible and thus culturally adaptable and especially suitable for clients in non-Western societies. One important aspect for its use in the Mekong I Project is the ease in which knowledge transfer – training, can take place. This enables broad dissemination in severely affected countries and the treatment of a vast number of trauma survivors in a safe and effective way.

#### Lack of Research about Stabilisation

Even though Phase Orientated approaches and stabilisation techniques are commonly used in therapeutic practise and recommended by the ISTSS guidelines for the treatment of complex PTSD, there is still a lack of evidence, especially compared to the numerous studies supporting evidence of TF-CBT and EMDR therapy. A group of researchers and therapists even published a letter to the editor criticizing the current ISTSS guideline that recommends the phase orientated approach [37]. They argue that with the lack of evidence of the effectiveness of a preparation/stabilisation phase and the effectiveness of stabilisation in general, only the evidence-based trauma focussed approaches like TF-CBT and EMDR therapy should be recommended in the guideline instead of a phase orientated approach.

The frequent use of trauma stabilisation techniques in the Mekong Project I, as this paper will demonstrate, offers the opportunity to better understand the impact of this approach as a distinct intervention, in an area where there is a scarcity of research data currently.

## Method

### The Data of the Mekong I Project

#### Therapists and Data Collection of the Mekong I Project

During the Mekong I Project 37 therapists were trained - 9 from Cambodia, 12 Indonesia, 16 Thailand. The professional background of the trainees – 32 Psychologists and 5 Psychiatrists, all of which had previous psychotherapy training. Of the 37 trainees – 21 Female, 16 Male with an age range of 29 to 62 years, and mean age of 39.48 years. All the therapists where trained on a pro-bono basis. However, a condition was that they were expected to offer pro-bono treatment back in their respective communities and collect clinical data about their clients. Data collection also included psychometrics and diagnostic measures. Ethical approval for the study was granted from the University of Worcester (UK) and was adopted in each the three countries.

The clients were service seekers that voluntarily participated in trauma treatment with each informed about the possibility to withdraw their participation at any point – in accordance with ethics approval guidelines. All in all, the demographic, diagnostic and treatment data of 4799 clients was documented and subsequently analysed. Data was first collected, via Microsoft Excel Spread Sheet, in each country for the period of the research study – 3 years, before being put together into a collective data set ready for retrospective analysis using post inclusion criteria.

#### Data of Clients of the Mekong I Project

The centralised Microsoft Excel sheet was transferred into SPSS version 24. The recorded data of the 37 therapists had to be further structured and partly recorded to create a coherent data file. The result is that this large data set contained over 2000 variables of the 4799 clients who took part in the study. The profiles were as follows:

- Indonesia (*n* = 2363, 49.2%), Cambodia (*n* = 1483, 30.9%), Thailand (*n* = 953, 19.9%)
- Type of Client: Adults (*n* = 2561, 53.4%), Children/adolescents (*n* = 2238, 46.6%)
- Female (*n* = 2709, 56.4%), Male clients (*n* = 2057, 42.9%), Transsexual (*n* = 33, 0.7%)

#### Preparation of the Data

Most of the data collected is mainly quantitative, however some qualitative material was also captured. About 30% of the qualitative data was documented using indigenous language of the respective therapists and then translated by local linguistic experts into English. The qualitative data was analysed using a program for semantic analysis by Braun and Clarke [38]. However only the quantitative date will be presented in this paper.

### Analysis for this Article

#### The Included Data -Inclusion Criteria and Description of Clients and Therapies

This article will only present the research findings from the adult client group who received trauma stabilisation interventions only. For the study, Trauma Stabilisation is defined as including resource interventions, grounding techniques, comprehensive history taking, trauma preparation, trauma mapping, trauma case conceptualisation and psychoeducation. For each client each therapist wrote a treatment outline containing the number of sessions, setting and duration of each session and indicated the specific stabilisation/confrontation interventions used each session. Only clients were included in the analysis who had received no trauma confrontation intervention at all and at least one intervention of trauma stabilisation. Treatment fidelity was ensured through active clinical supervision and through supervised self-experience teaching and learning sessions. Using this inclusion criteria data was analysed from *n* = 1358 clients. For detailed demographic information please view Table 1. The mean amount of sessions, for the included data, was 4.31 (SD 3.24, range 1 to 23). The mean number of Trauma Stabilisation interventions used was 7.23 (SD 5.50, range 1 to 64).

**Table 1:** Demographic information and number of sessions/trauma stabilisation techniques for the clients of the whole data set and respectively for all steps of inclusion

Characteristics		Inclusion criteria for analysis			
	Mekong data	only adults, stabilisation	Pre- & post-measures	HTQ diagnosis pre-treatment	
				DSM-V PTSD	ICD-11 PTSD
N	4799 (100%)	1358 (100%)	365 (100%)	197 (100%)	164 (100%)
Country					
Cambodia	1483 (30.9%)	654 (48.2%)	206 (56.4%)	128 (65.0%)	98 (59.8%)
Indonesia	2363 (49.2%)	414 (30.5%)	78 (21.4%)	46 (23.4%)	46 (28.0%)
Thailand	953 (19.9%)	290 (21.4%)	81 (22.2%)	23 (11.7%)	20 (12.2%)
Client group					
Adults	2561 (53.4%)	1358 (100%)	365 (100%)	197 (100%)	164 (100%)
Children/Adolescents	2238 (46.6%)	–	–	–	–
Gender					
Male	2057 (42.9%)	468 (34.5%)	103 (28.2%)	46 (23.4%)	37 (22.6%)
Female	2709 (56.4%)	885 (65.2%)	258 (70.7%)	147 (74.6%)	124 (75.6%)
Transgender	33 (0.7%)	5 (0.4%)	4 (1.1%)	4 (2.0%)	3 (1.8%)
Age (Mean (SD))	23.39 (12.27)	31.69 (12.02)	34.52 (11.81)	36.76 (11.01)	36.93 (11.07)
Therapies					
Mean N Sessions (SD)	5.4 (4.7)	4.3 (3.2)	6.7 (3.9)	7.7 (3.9)	7.6 (3.8)
Mean N stabilisation techniques (SD)	9.5 (10.1)	7.2 (5.5)	11.0 (6.6)	10.5 (7.1)	10.0 (6.9)

#### Diagnostic Measures

Two specific psychometrics were used. For assessing the PTSD diagnosis, the Harvard Trauma Questionnaire (HTQ - 40 items), and the Hopkins Symptoms Checklist Questionnaire (HSCL-25 items) to assess Anxiety and Depression – pre- and post-treatment. Both scales were deliberately chosen as they have international application and validation and were subsequently available in language versions in Indonesian, Khmer and Thai. Therapists were trained and data evaluated strictly according to the manual “Measuring Trauma Measuring Torture” of the Harvard Program in Refugee Trauma [39]. Internal consistency (Cronbach’s α) at baseline of the HTQ and HSCL-25 in our sample was 0.96 and 0.94, respectively.

#### Description of Reported Results

The publication focuses on PTSD diagnosis in adult populations in Indonesia, Cambodia and Thailand however, further outcomes will be reported in future publications.

For the clients, the following results can be reported:

- Remission rates: the PTSD scale of the HTQ includes items reflecting the DSM-V and the forthcoming ICD-11 criteria for PTSD. A study investigating the impact of the changes to diagnostic criteria for PTSD in DSM-V and the proposed changes in ICD-11 found that while there is an overlap of the PTSD diagnoses, each identified a proportion of people with PTSD which the other system did not [40]. As different study populations are defined depending on the diagnostic classification system used, the comparison between studies using different diagnosis systems is hindered. To prevent this, for the analysis DSM-V as well as ICD-11 PTSD is reported. PTSD status was calculated using the item mapping for the DSM-V and ICD-11 models of PTSD as suggested by Hyland [41]. Criterion A (traumatic stressor) was established with a detailed list of Traumatic Events. PTSD is diagnosed via DSM-V criteria when a client scores 3 or 4 in (i) at least one of the four intrusion symptoms (criterion B), (ii) at least one of the two avoidance symptoms (criterion C), (iii) at least two of the seven symptoms regarding negative alternations in cognitions and mood (abbreviation Neg.Alt. Cogn&Mood, criterion D), and (iv) at least two of the five arousal symptoms (criterion E). PTSD is diagnosed via the purposed ICD-11 criteria when a client scores 3 or 4 in (i) at least one of the two re-experiencing symptoms, (ii) at least one of the two sense of threat symptoms and (iii) at least one of the two avoidance symptoms. Patients no longer fulling these criteria of DSM-V/ICD-11 PTSD after treatment are regarded as remitted.

Additionally to Remission rates, the Treatment Effect can also be reported. The treatment effect is calculated as the reduction of fulfilled PTSD diagnoses and the syndrome reduction due to Trauma Stabilisation interventions. Only clients were included in the analysis, where pre- and post-measures of the HTQ were available, 365 clients meet this criterion.

#### Inclusion Criterium Fulfilment of PTSD Diagnosis

Pre-treatment 197 (54.0%) of the included 365 clients fulfilled the diagnosis of PTSD via DSM-V criteria and 164 (44.9%) via ICD-11 criteria. For the following analysis, only clients fulfilling the respective diagnosis pre-treatment (DSM-V/ICD-11 PTSD) are included. In a later section, the results for all included clients regardless their PTSD diagnosis pre-treatment are reported.

#### Statistical Analysis

All data was analysed using SPSS version 24. The pairwise comparisons of the binary variables (remission rates of PTSD diagnoses and PTSD criteria) were calculated via McNemar tests. The pairwise comparisons of the number of criteria fulfilled of the respective PTSD diagnoses and of the number of symptoms of the respective DSM-V/ICD-11 criteria were calculated via dependent t-tests. From the t-tests the effect sizes *r* and Hedges’s g_av_ were calculated. As Cohen’s d_z_ is argued to be an overestimation of the effect size of correlated samples and Cohen’s d_av_ is positively biased, Hedges’s corrected and recommended Hedges’s g_av_ is reported [42, 43]. The *a*-level was Bonferroni adjusted for multiple comparisons (*a* = 0.0028 = 0.05/18; PTSD DSM-V and ICD-11 remission, Number of criteria of DSM-V and of ICD-11 PTSD, criteria of PTSD (DSM-V: Intrusions, Neg.Alt. Cogn&Mood, Arousal, Avoidance, ICD-11: Re-experiencing, Sense of threat, Avoidance), Number of symptoms of the five DSM-V and the four ICD-11 PTSD criteria).

## Results

**Fig. 1:**
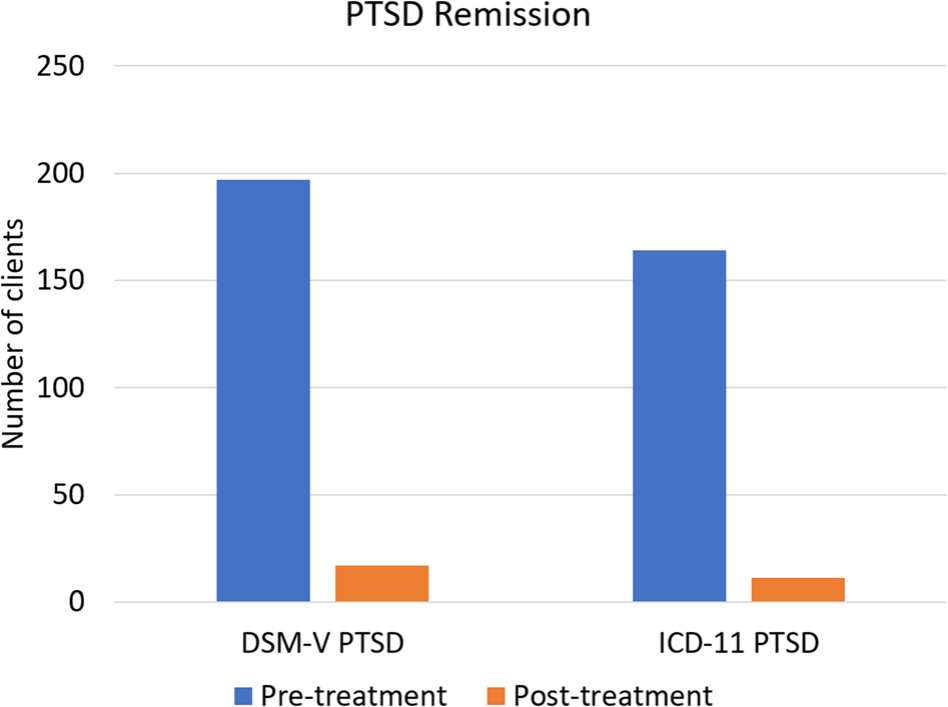
PTSD Remission after trauma stabilisation treatment: Number of clients fulfilling DSM-V/ICD-11 PTSD prior treatment (blue/black bars) and Number of clients still fulfilling DSM-V/ICD-11 PTSD after treatment (orange/ grey bars)

PTSD remission rates were 91.4% for DSM-V and 93.3% for ICD-11 diagnosis as highlighted in Fig. 1. McNemar tests determined that there was a significant difference of the proportion of clients with PTSD diagnosis pre- and post-treatment, (*p* < 0.00001 for both DSM-V and ICD-11 PTSD), for further details please view Table 2. On average, clients fulfilled less DSM-V PTSD criteria after the trauma stabilisation treatment (*M* = 1.97, *SD* = 1.387) than before treatment (*M* = 5.0, *SD* = 0.00). This difference, 3.025, BCa 95% CI [2.831, 3.220] was significant *t*(196) = 30.623, *p* < 0.001, and represented a large-sized effect, *r* = 0.910 and Hedges’s g_av_ = 3.078, BCa 95% CI [2.875, 3.271]. The CL effect size indicates that after controlling for individual differences, the likelihood that a person scores higher pre-treatment than after treatment is 98.6%. On average, clients fulfilled less ICD-11 PTSD criteria after treatment (*M* = 1.54, *SD* = 0.92) than before treatment (*M* = 4.0, *SE* = 0.00). This difference, 2.457, BCa 95% CI [2.316, 2.599] was significant *t*(163) = 34.363, *p* < 0.001, and represented a large-sized effect, *r* = 0.937 and Hedges’s g _av_ = 3.780, BCa 95% CI [3.559, 3.994]. The CL effect size indicates that after controlling for individual differences, the likelihood that a person scores higher pre-treatment than after treatment is 99.6%. These results are also highlighted in Table 3.

**Table 2:** Remission of (respective DSM-V/ICD-11) PTSD diagnosis after trauma stabilisation treatment

HTQ	Number of participants		Remission rate	McNemar test	
	Pre-treatment	Post-treatment		Test statistic^a^	*p*
DSM-V PTSD	197 (100%)	17 (8.6%)	91.4%	178.006	< 0.00001
ICD-11 PTSD	164 (100%)	11 (6.7%)	93.3%	151.007	< 0.00001

^a^Chi-Square, continuity corrected

**Table 3:** Remission of PTSD criteria after trauma stabilisation treatment: Pairwise comparisons of the mean number of fulfilled criteria for DSM-V/ICD-11 PTSD before and after the trauma stabilisation treatment for clients fulfilling DSM-V/ICD-11 PTSD pre-treatment

HTQ	Raw mean (standard deviation)		*t*	Bootstrap *p*	Hedges’s g _av_^a^ [BCa 95% CI]	*r*
Nr. Of criteria	Pre-treatment	Post-treatment				
DSM-V PTSD^b^	5.00 (0.0)	1.97 (1.387)	30.623	< 0.001	3.08 [2.88, 3.27]	0.910
ICD-11 PTSD^c^	4.00 (0.0)	1.54 (0.916)	34.363	< 0.001	3.78 [3.56, 3.99]	0.937

^a^Hedges’s g _av_ is used as a corrected effect size to the biased Cohen’s d_av_, ^b^0–5 criteria of DSM-V PTSD possible, ^c^0–4 criteria of ICD-11 PTSD possible

Remission rates for DSM-V PTSD and ICD-11 PTSD criteria ranged between 72.1 and 86.0%. McNemar tests determined that there were significant differences of the proportion of clients with PTSD criteria pre- and post-treatment, (*p* < 0.00001 for both DSM-V and ICD-11 PTSD criteria), for further details please view Table 4. On average, clients fulfilled less symptoms of the respective DSM-V PTSD criteria after treatment than before treatment. These differences were significant (*p* < 0.001 for all symptom criteria) and represented large-sized effects (*r* between 0.865 and 0.914 and Hedges’s g _av_ between 2.50 and 3.09), for further details please view Table 5. The CL effect sizes indicate that after controlling for individual differences, the likelihood that a person scores higher pre-treatment than after treatment in symptoms of DSM-V PTSD intrusions is 95.8%, in symptoms of DSM-V PTSD Neg. Alt. Cogn&Mood 97.8%, in symptoms of DSM-V PTSD Arousal 98.8%. and in symptoms of DSM-V PTSD Avoidance 97.9%. On average, clients fulfilled less symptoms of the respective ICD-11 PTSD criteria after treatment than before treatment. These differences were significant (*p* < 0.001 for all symptom criteria) and represented large-sized effects (*r* between 0.892 and 0.910 and Hedges’s g _av_ between 2.88 and 3.25). The CL effect sizes indicate that after controlling for individual differences, the likelihood that a person scores higher pre-treatment than after treatment in symptoms of ICD-11 PTSD re-experiencing is 98.5%, in symptoms of ICD-11 PTSD Sense of threat 97.6% and in symptoms of ICD-11 PTSD Avoidance 98.6%.

**Table 4:** Remission of DSM-V and of ICD-11 PTSD criteria after the trauma stabilisation treatment for clients fulfilling DSM-V/ICD-11 PTSD pre-treatment

HTQ	Number of participants		Remission rate	McNemar test	
	Pre-treatment	Post-treatment		Test statistic^a^	*p*
DSM-V PTSD^b^					
Intrusions	197 (100%)	50 (25.4%)	74.6%	145.007	< 0.00001
Neg.Alt. cogn.&mood	197 (100%)	46 (23.4%)	76.6%	149.007	< 0.00001
Arousal	197 (100%)	55 (27.9%)	72.1%	140.007	< 0.00001
Avoidance	197 (100%)	41 (20.8%)	79.2%	154.006	< 0.00001
ICD-11 PTSD^b^					
Re-experiencing	164 (100%)	23 (14.0%)	86.0%	139.007	< 0.00001
Sense of threat	164 (100%)	34 (20.7%)	79.3%	128.008	< 0.00001
Avoidance	164 (100%)	32 (19.5%)	80.5%	130.008	< 0.00001

^a^Chi-Square, continuity corrected

^b^except criterion A where no remission is possible

**Table 5:** Remission from PTSD symptoms after trauma stabilisation treatment: Pairwise comparisons of the mean number of fulfilled symptoms of the respective criterium for DSM-V/ICD-11 PTSD pre- and post-treatment for clients fulfilling DSM-V/ICD-11 PTSD pre-treatment

HTQ	Raw mean (standard deviation)		*t*	Bootstrap *p*	Hedges’s g _av_^a^ [BCa 95% CI]	*r*
Nr. Of symptoms	Pre-treatment	Post-treatment				
DSM-V PTSD						
Intrusions^b^	3.06 (1.041)	0.50 (1.003)	24.171	< 0.001	2.50 [2.30, 2.68]	0.865
Neg.Alt. cogn.&mood^c^	5.17 (1.575)	0.86 (1.515)	28.285	< 0.001	2.78 [2.58, 3.00]	0.896
Arousal^d^	4.335 (0.886)	0.93 (1.272)	31.568	< 0.001	3.09 [2.91, 3.28]	0.914
Avoidance^e^	1.79 (0.407)	0.28 (0.598)	28.607	< 0.001	2.94 [2.75, 3.11]	0.900
ICD-11 PTSD						
Re-experiencing^f^	1.59 (0.493)	0.18 (0.469)	27.683	< 0.001	2.92 [2.72, 3.13]	0.908
Sense of threat^f^	1.77 (0.423)	0.28 (0.592)	25.228	< 0.001	2.88 [2.67, 3.09]	0.892
Avoidance^e^	1.84 (0.372)	0.26 (0.574)	28.044	< 0.001	3.25 [3.00, 3.48]	0.910

^a^Hedges’s g _av_ is a corrected effect size to the biased Cohen’s d_av_

^b^0–4 symptoms of DSM-V PTSD Intrusions possible

^c^0–8 symptoms of DSM-V PTSD Neg.Alt. Cogn&Mood possible

^d^0–5 symptoms of DSM-V PTSD Arousal possible

^e^0–2 symptoms of DSM-V/ICD-11 PTSD Avoidance possible

^f^0–2 symptoms of ICD-11 PTSD Re-experiencing/Sense of threat possible

### Results for the Entire Range of PTS Problems

**Fig. 2:**
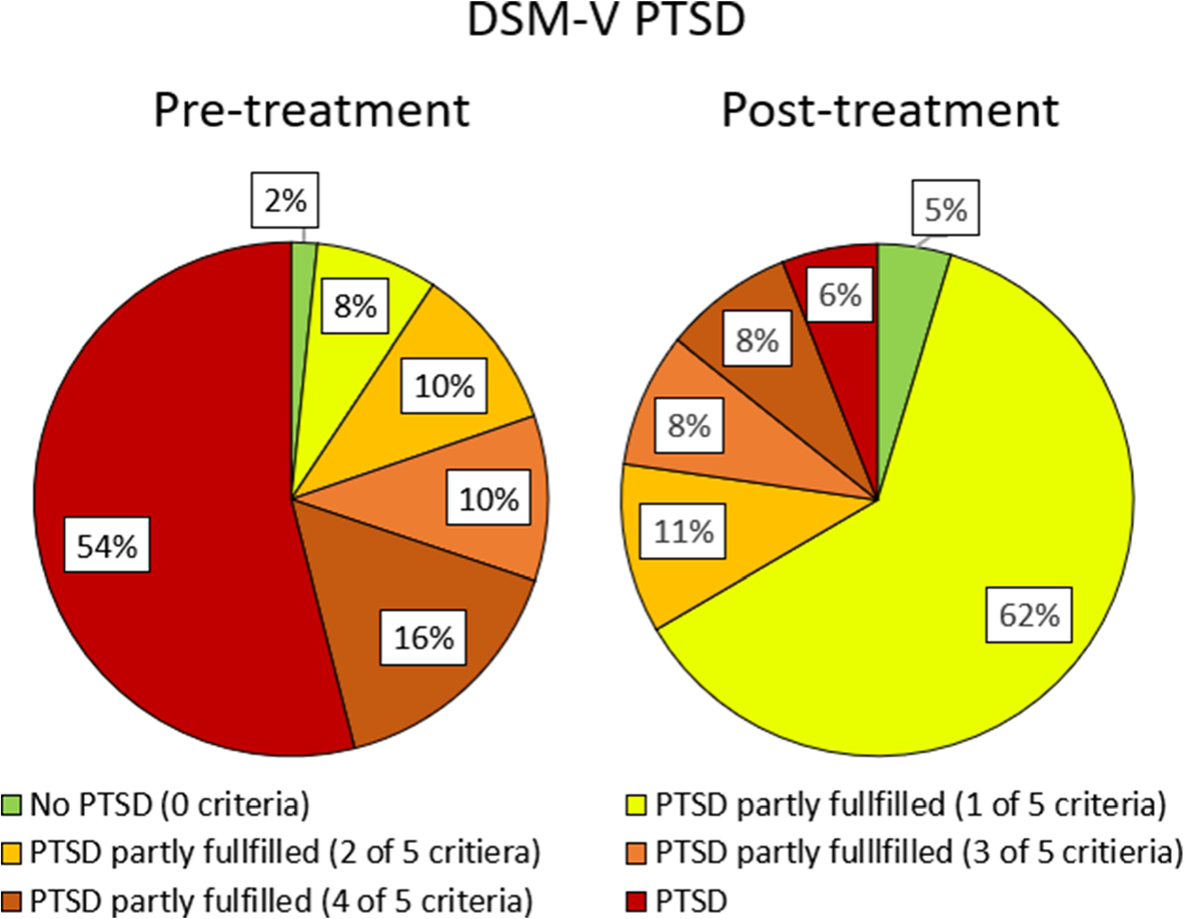
Distribution of clients fulfilling DSM-V PTSD (black/red), partly fulfilling PTSD (1 up to 4 of 5 DSM-V PTSD criteria fulfilled, 4 ranges of grey/orange, lighter colours represent-ing less criteria), no PTSD (white/green) before and after trauma stabilisation treatment

**Fig. 3:**
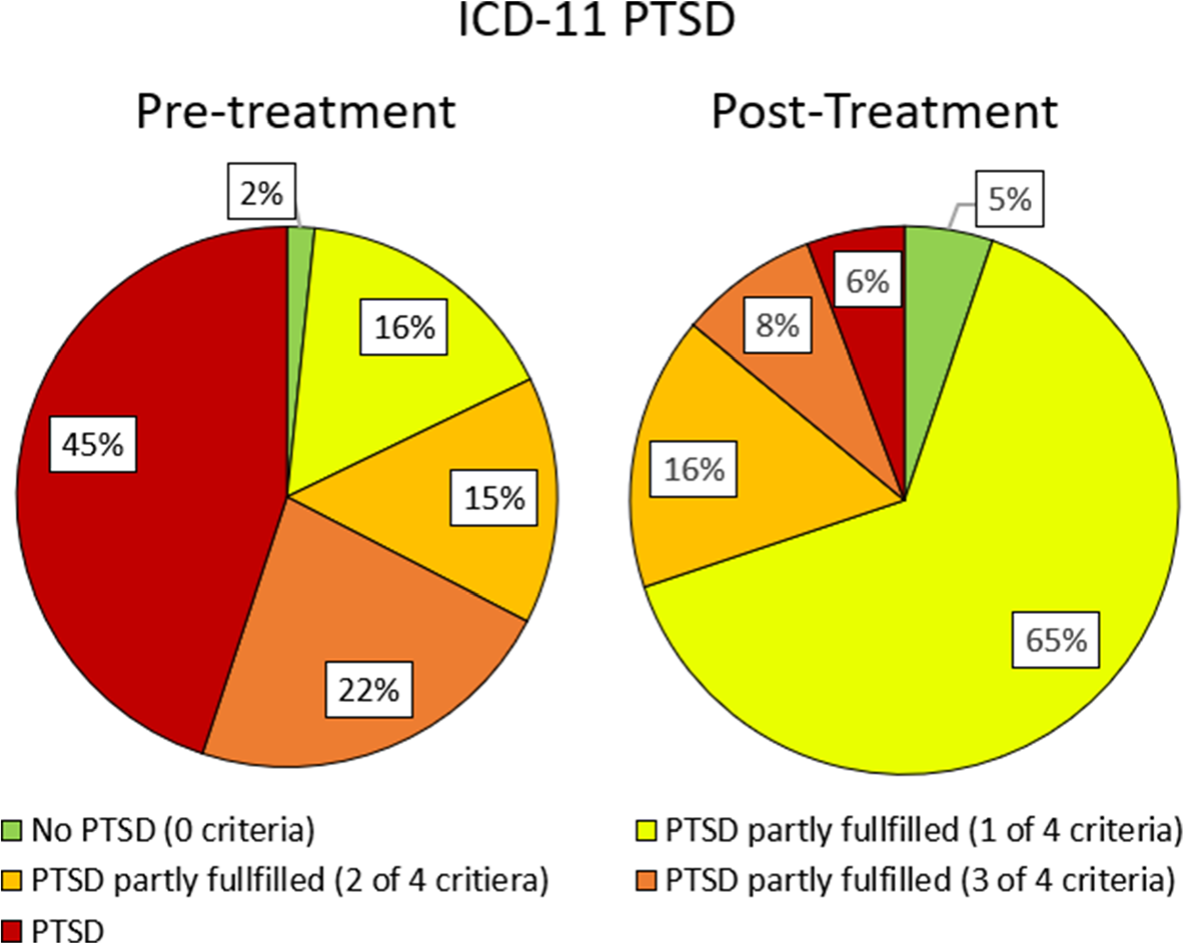
Distribution of clients fulfilling ICD-11 PTSD (red/dark grey), partly fulfilling PTSD (1 (orange/grey) or 2 (yellow/light grey) of 3 ICD-11 PTSD criteria fulfilled), no PTSD (green/white) before and after trauma stabilisation treatment

Additionally to the treatment effect of clients with DSM-V/ICD-11 PTSD pre-treatment, the treatment effect for subclinical clients and ‘normal’ scoring clients can be reported. In this section, the analysis of all included clients (365 clients) regardless of their diagnosis before treatment is reported. The distribution of fulfilled PTSD criteria pre- and post-treatment is shown in Figs. 2 and 3.

**Figs 4 and 5:**
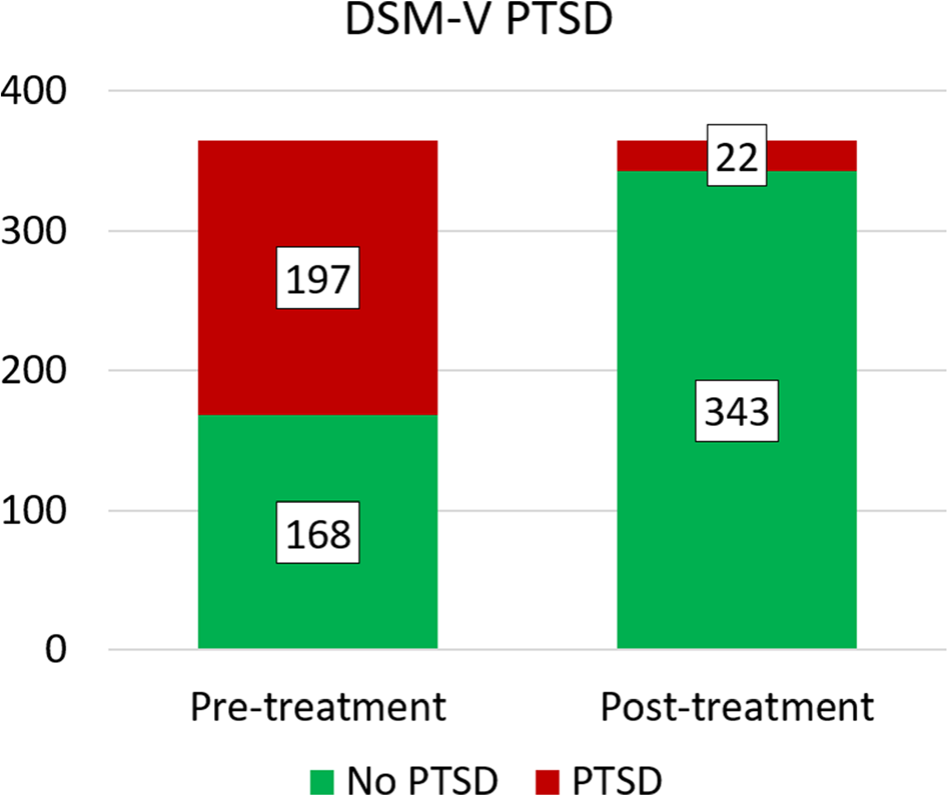

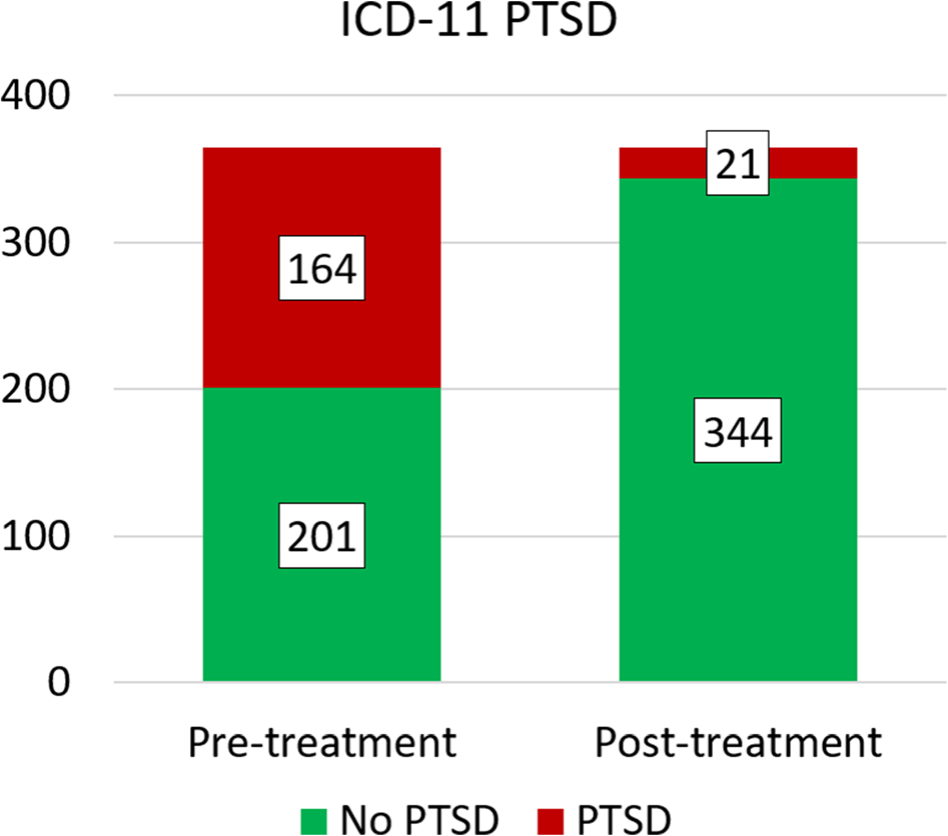
Proportion of clients with PTSD (red/black) and without PTSD (green/grey) diagnoses before and after trauma stabilisation for DSM-V and ICD-11 PTSD respectively

Six clients (1.6%) did not fulfil any criteria of DSM-V PTSD. For 83.3% this didn’t change after treatment, 1 client was subclinical after treatment (fulfilling three criteria of DSM-V PTSD). 162 clients (44.4%) were subclinical prior treatment fulfilling one (28 clients), two (38 clients), three (38 clients) or four (58 clients) DSM-V PTSD criteria prior treatment. For this subclinical client group 12 clients remised (no criteria of DSM-V PTSD after treatment), 104 clients (64.2%) improved, but stayed subclinical, 7 clients (4.3%) deteriorated, but remained subclinical and 5 clients (3.1%) were diagnosed with DSM-V PTSD still after treatment.

6 clients (1.6%) did not fulfil any criteria of ICD-11 PTSD pre-treatment. For all 6 clients, this didn’t change after treatment. 195 clients (53.2%) were subclinical prior treatment fulfilling one (59 clients), two (54 clients) or three (82 clients) ICD-11 PTSD criteria prior treatment. For this subclinical client group 13 clients remised (no criteria of ICD-11 PTSD after treatment), 93 clients (47.7%) improved, but stayed subclinical, 9 clients (4.6%) deteriorated, but remained subclinical and 10 clients (5.1%) were diagnosed with ICD-11 PTSD after treatment.

With 5 clients developing DSM-V PTSD and 10 clients developing ICD-11 PTSD during the time of the treatment, the analysed treatment effect might change, if the whole client group (no, subclinical, clinical PTSD) is included. Thus, all analyses were calculated once more with all clients regardless their diagnoses pre-treatment (*N* = 365 clients). The proportion of clients with and without PTSD before and after treatment is highlighted in Figs. 4 and 5.

McNemar tests determined that there was a significant difference of the proportion of clients with PTSD diagnosis pre- and post-treatment, (*p* < 0.00001 for both DSM-V and ICD-11 PTSD). The odd of remission of DSM-V PTSD is 36 times greater than the risk of getting the DSM-V PTSD diagnoses after treatment. The odd of remission of ICD-11 PTSD is 15.3 times greater than the risk of getting the ICD-11 PTSD diagnoses after treatment. For further details please view Table 6. On average, clients fulfilled less DSM-V PTSD criteria after treatment (*M* = 1.72, *SD* = 1.299) than before treatment (*M* = 3.93, *SD* = 1.419). This difference, 2.214, BCa 95% CI [2.046, 2.384] was significant *t*(364) = 25.474, *p* < 0.001, and represented a large-sized effect, *r* = 0.800 and Hedges’s g _av_ = 1.621, BCa 95% CI [1.499, 1.750]. The CL effect size indicates that after controlling for individual differences, the likelihood that a person scores higher pre-treatment than after treatment is 90.8%. On average, clients fulfilled less ICD-11 PTSD criteria after treatment (*M* = 1.45, *SD* = 0.93) than before treatment (*M* = 2.93, *SD* = 1.18). This difference, 1.48, BCa 95% CI [1.354, 1.611] was significant *t*(365) = 20.948, *p* < 0.001, and represented a large-sized effect, *r* = 0.739 and Hedges’s g _av_ = 1.391, BCa 95% CI [1.273, 1.515]. The CL effect size indicates that after controlling for individual differences, the likelihood that a person scores higher pre-treatment than after treatment is 86.3%. These results are also shown in Table 7.

**Table 6:** Remission of PTSD diagnosis after trauma stabilisation treatment: Pairwise comparisons of PTSD diagnosis (via DSM-V and ICD-11 respectively) before and after trauma stabilisation treatment for all 365 clients

HTQ	Number of participants		McNemar		OR remission/ risk
	Pre-treatment	Post-treatment	Test statistic^a^	*p*	
DSM-V PTSD	197 (54.0%)	22 (6.0%)	163.654	< 0.00001	36
ICD-11 PTSD	164 (44.9%)	21 (5.8%)	123.706	< 0.00001	15.3

^a^Chi-Square, continuity corrected

**Figs 6 and 7:**
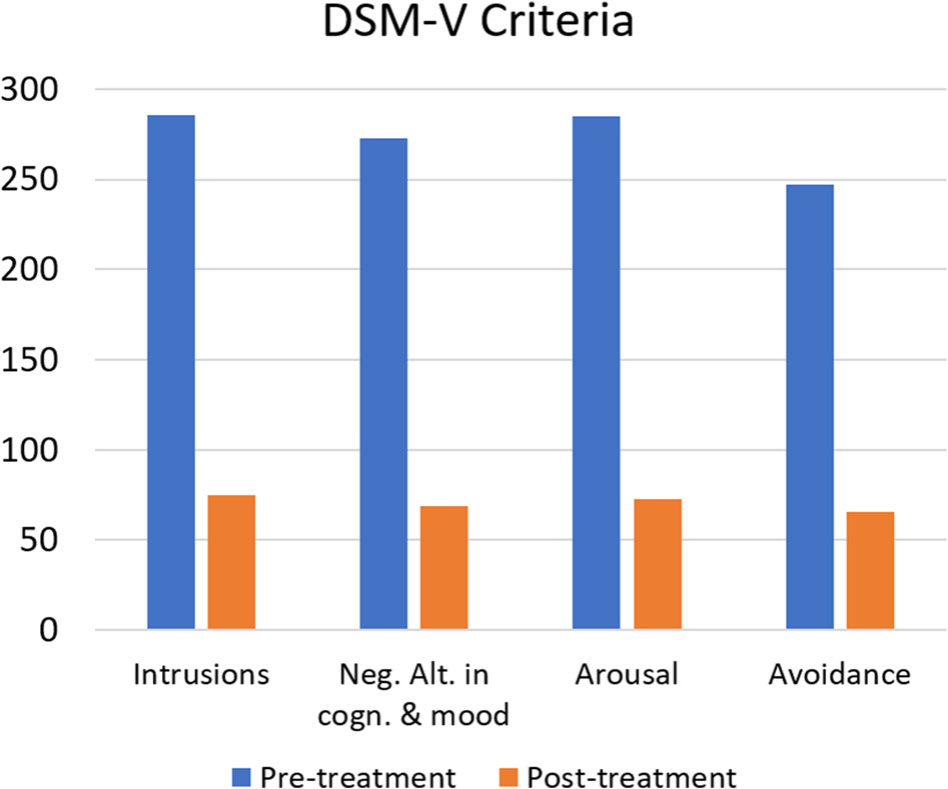

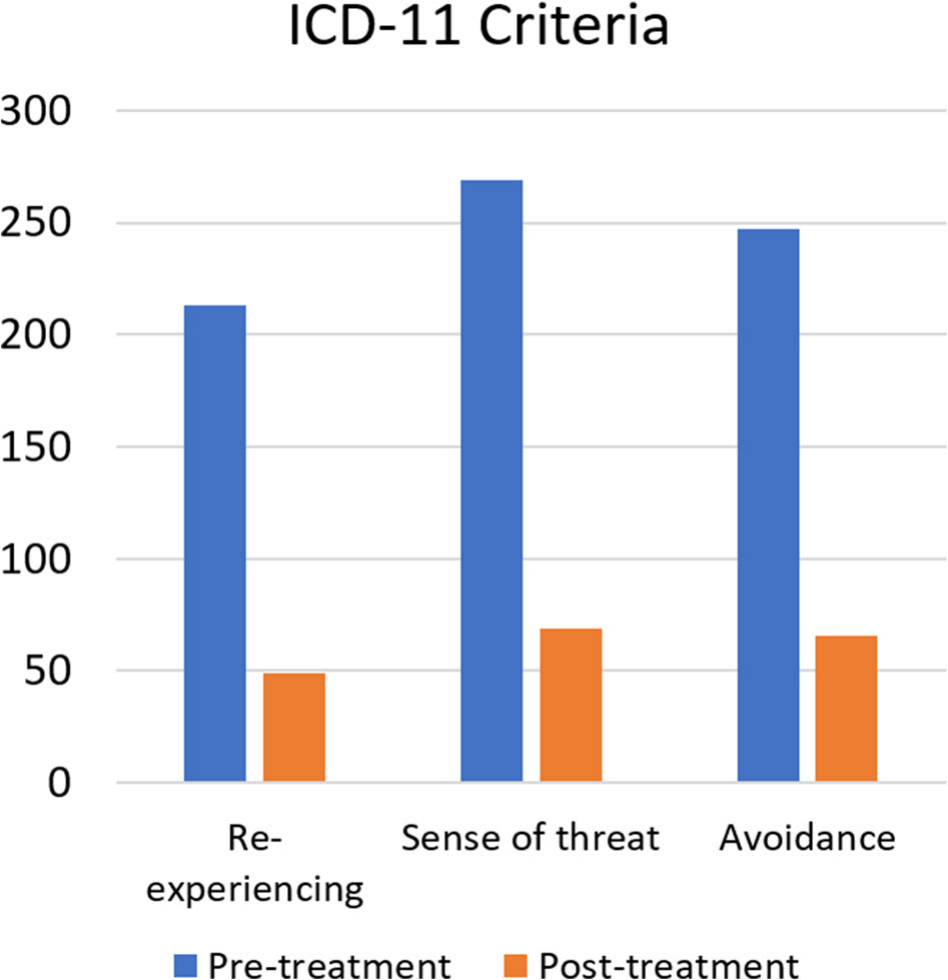
Remission of PTSD criteria after trauma stabilisation treatment: Number of participants fulfilling DSM-V/ICD-11 PTSD criteria prior treatment (blue/black bars) and after treatment (orange/ grey bars)

**Table 7:** Remission of PTSD criteria after trauma stabilisation treatment: Pairwise comparisons of the mean number of fulfilled DSM-V/ICD-11 PTSD criteria before and after trauma stabilisation treatment for all 365 clients

HTQ	Raw mean (standard deviation)		*t*	*p*	Hedges’s g _av_^a^ [95% CI]	*r*
Nr. Of criteria	Pre-treatment	Post-treatment				
DSM-V PTSD^b^	3.93 (1.419)	1.72 (1.299)	25.474	< 0.001	1.62 [1.50, 1.75]	0.800
ICD-11 PTSD^c^	2.93 (1.179)	1.45 (0.929)	20.948	< 0.001	1.39 [1.27, 1.51]	0.739

^a^Hedges’s g _av_ is used as a corrected effect size to the biased Cohen’s d_av_

^b^ 0–5 criteria of DSM-V PTSD possible

^c^0–3 criteria of ICD-11 PTSD possible

**Figs 8 and 9:**
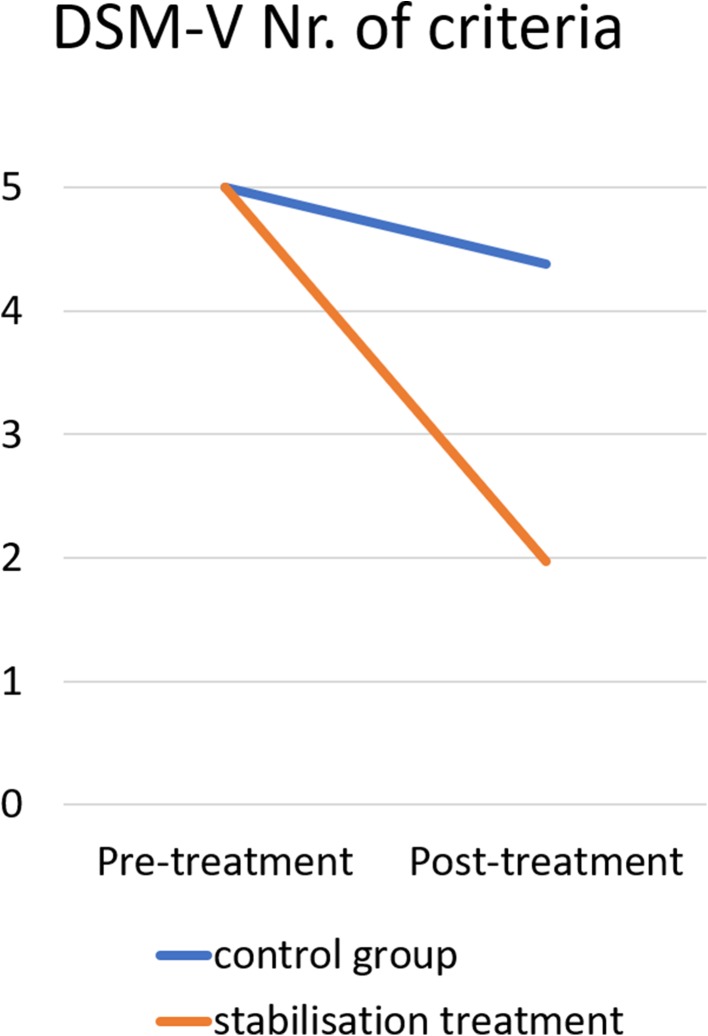

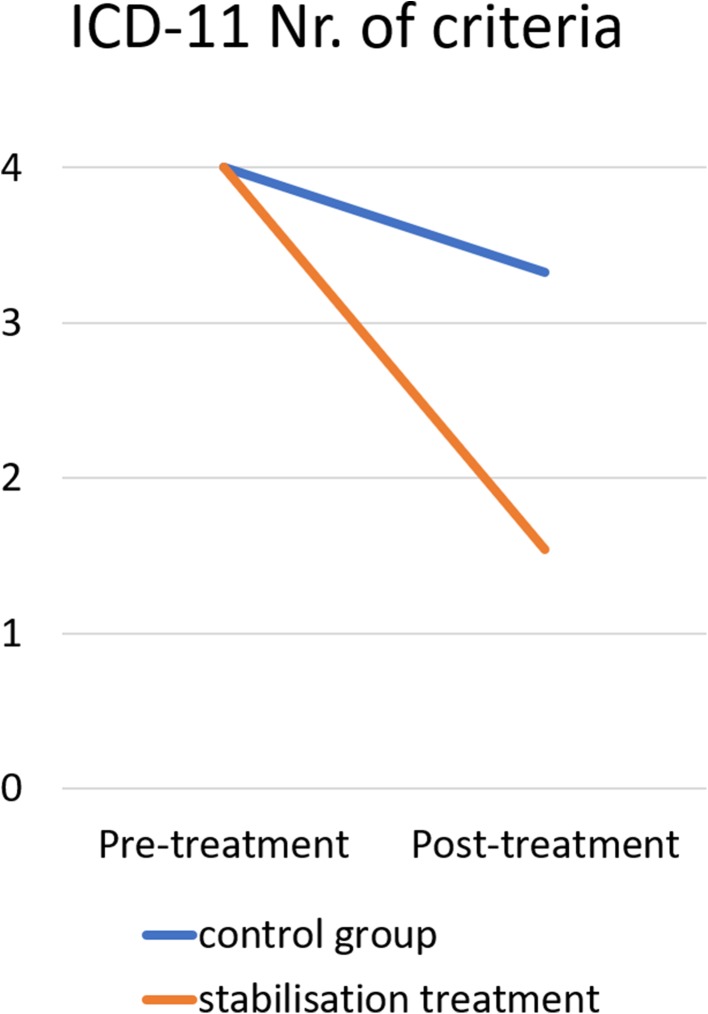
Remission of the five DSM-V PTSD criteria and of the four ICD-11 PTSD criteria for the trauma stabilisation treatment group (orange/grey line) and for the waiting-list control group (blue/black line)

On average, clients fulfilled less symptoms of the respective DSM-V PTSD criteria after treatment than before treatment. These differences were significant (*p* < 0.00001 for all symptom criteria) and represented large-sized effects (*r* between 0.672 and 0.789 and Hedges’s g _av_ between 1.25 and 1.63). The CL effect sizes indicate that after controlling for individual differences, the likelihood that a person scores higher pre-treatment than after treatment in symptoms of DSM-V PTSD intrusions is 87.1%, in symptoms of DSM-V PTSD Neg.Alt. Cogn&Mood 86.2%, in symptoms of DSM-V PTSD Arousal 90.0%. and in symptoms of DSM-V PTSD Avoidance 81.9%. On average, clients fulfilled less symptoms of the respective ICD-11 PTSD criteria after treatment than before treatment. These differences were significant (*p* < 0.001 for all symptom criteria) and represented large-sized effects (*r* between 0.672 and 0.789 and Hedges’s g _av_ between 1.25 and 1.63). The CL effect sizes indicate that after controlling for individual differences, the likelihood that a person scores higher pre-treatment than after treatment in symptoms of ICD-11 PTSD re-experiencing is 78.4%, in symptoms of ICD-11 PTSD Sense of threat 83.8%. and in symptoms of DSM-V PTSD Avoidance 81.8%. For detailed results please see Tables 8 and 9. The remission of PTSD criteria after treatment is highlighted in Figs. 6 and 7.

**Table 8:** Pairwise comparisons of DSM-V and ICD-11 PTSD criteria fulfilled before and after trauma stabilisation treatment for all 365 clients

HTQ	Number of participants		McNemar test		OR _remission/risk_
	Pre-treatment	Post-treatment	Test statistic^a^	*P*	
DSM-V PTSD					
Intrusions	286 (78.4%)	75 (20.5%)	189.270	< 0.00001	20.18
Neg.Alt. cogn.&mood	273 (74.8%)	69 (18.9%)	189.032	< 0.00001	30.14
Arousal	285 (78.1%)	73 (20.0%)	202.368	< 0.00001	54
Avoidance	247 (67.7%)	66 (18.1%)	150.698	< 0.00001	11.65
ICD-11 PTSD					
Re-experiencing	213 (58.4%)	49 (13.4%)	130.240	< 0.00001	9.2
Sense of threat	265 (72.6%)	69 (18.9%)	176.042	< 0.00001	20.6
Avoidance	247 (67.7%)	66 (18.1%)	150.698	< 0.00001	11.65

^a^Chi-Square, continuity corrected

**Table 9:** Remission of PTSD symptoms after trauma stabilisation treatment: Pairwise comparisons of the mean number of fulfilled symptoms of the respective criterium for DSM-V/ICD-11 PTSD before and after trauma stabilisation treatment for all 365 clients

HTQ	Raw mean (standard deviation)		*t*	bootstrap *p*	Hedges’s g _av_^a^ [BCa 95% CI]	*r*
Nr. Of symptoms	Pre-treatment	Post-treatment				
DSM-V PTSD						
Intrusions^b^	2.12 (1.512)	0.41 (0.932)	19.203	< 0.001	1.56 [1.40, 1.73]	0.709
Neg.Alt. cogn.&mood^c^	3.59 (2.411)	0.73 (1.414)	20.873	< 0.001	1.44 [1.30, 1.60]	0.738
Arousal^d^	3.145 (1.763)	0.72 (1.153)	24.489	< 0.001	1.63 [1.50, 1.76]	0.789
Avoidance^e^	1.16 (0.885)	0.24 (0.552)	17.356	< 0.001	1.25 [1.09, 1.39]	0.672
ICD-11 PTSD						
Re-experiencing^f^	0.89 (0.843)	0.16 (0.445)	14.900	< 0.001	1.08 [0.93, 1.20]	0.615
Sense of threat^f^	1.17 (0.831)	0.24 (0.543)	18.723	< 0.001	1.32 [1.18, 1.45]	0.700
Avoidance^e^	1.16 (0.885)	0.24 (0.552)	17.356	< 0.001	1.24 [1.10, 1.38]	0.672

^a^Hedges’s g _av_ is a corrected effect size to the biased Cohen’s d_av_

^b^0–4 symptoms of DSM-V PTSD Intrusions possible

^c^0–8 symptoms of DSM-V PTSD Neg.Alt. Cogn&Mood possible

^d^0–5 symptoms of DSM-V PTSD Arousal possible

^e^0–2 symptoms of DSM-V/ICD-11 PTSD Avoidance possible

^f^0–2 symptoms of ICD-11 PTSD Re-experiencing/Sense of threat possible

### Comparison to Control Group

To put the extremely high effect sizes in relation, the treatment effect was compared to a control group from a previously published study [35, 36] and aggregated. This was possible because it involved the same therapists from the Mekong I Project and included the same diagnostic tools and psychometrics. This made for a suitable comparison. The control group consisted of 55 clients (38 female clients, mean age 25.64 (SD 9.425)) who were waiting 5 weeks between their first and second diagnostic assessment.

45 clients (81.8%) of the control group fulfilled the criteria for DSM-V PTSD before waiting. The remission rate was 24.4% (vs. 91.4% remission rate for stabilisation treatment group). On average, clients fulfilled less DSM-V PTSD criteria after waiting (*M* = 4.38, *SD* = 1.451) than before treatment (*M* = 5.0, *SE* = 0.00). This difference, 0.622, BCa 95% CI [0.282, 1.057] was significant *t*(44) = 2.878, *p* = 0.025, and represented a medium-sized effect, *r* = 0.398 and Hedges’s g _av_ = 0.594, BCa 95% CI [0.270, 1.013]. The odd of remission of DSM-V PTSD is 2.2 times greater than the risk of getting the DSM-V PTSD diagnoses after waiting. 33 clients (60.0%) of the control group fulfilled the criteria for ICD-11 PTSD before waiting. The remis-sion rate was 33.3% for the control group (vs. 93.3% remission rate for stabilisation treatment group). On average, clients fulfilled less ICD-11 PTSD criteria after waiting (*M* = 3.33, *SD* = 1.216) than before treatment (*M* = 4.0, *SE* = 0.00). This difference, 0.667, BCa 95% CI [0.321, 1.081] was significant *t*(32) = 3.149, *p* = 0.025, and represented a medium-sized effect, *r* = 0.486 and Hedges’s g _av_ = 0.761, BCa 95% CI [0.365, 1.228]. The odd of remission of ICD-11 PTSD is 1.38 times greater than the risk of getting the ICD-11 PTSD diagnoses after waiting.

General linear model analyses were conducted to analyse differences in the PTSD remission between the two groups. There was a significant effect of the group, indicating that the remission of DSM-V PTSD criteria differs between stabilisation treatment and control group, *F*(1, 240) = 108.156, *p* < 0.00001, *r* = 0.557, η_p_^2^ = 0.311. This indicates that 31.03% of the variance between DSM-V PTSD pre- and posttreatment is explained by the treatment. The number needed to treat is 1.49. There was a significant effect of the group, indicating that the remission of ICD-11 PTSD criteria differs between stabilisation treatment and control group, *F*(1, 195) = 93.336, *p* < 0.00001, *r* = 0.569, η_p_^2^ = 0.324. This indicates that 32.38% of the variance between ICD-11 PTSD pre- and posttreatment is explained by the treatment. The number needed to treat is 1.67. The differences in remission are highlighted in Figs. 8 and 9.

## Discussion

### Prevalence Rates

#### Prevalence of PTSD

To our knowledge, such a large group of clients with trauma-related disorders has never been scrutinised so thoroughly anywhere in one of the three countries or in Southeast-Asia as whole. There are very high levels of PTSD in this service-seeking group of the clients. In the client group fulfilling all inclusion criteria (only stabilisation/psychoeducation, no confrontation, pre-and post-treatment measures) the prevalence rate of PTSD was 44.9% for ICD-11 PTSD (164 of 365 adults) and 54.0% for DSM-V PTSD (197 of 356 adults). In the client group fulfilling all inclusion criteria except post-diagnostic measures criteria (only stabilisation/ psychoeducation, no confrontation, pre-treatment measure) the prevalence rate was 42.4% for ICD-11 PTSD (382 of 901 adults) and 50.2% for DSM-V PTSD (452 of 901 adults). The prevalence of PTSD for all service-seeking adults with pre-treatment measurement is 47.5% for ICD-11 PTSD (947 of 1993 adults) and 54.8% for DSM-V PTSD (1092 of 1993 adults).

#### Prevalence of PTSD Syndrome Scales

The prevalence rates for the DSM-V and ICD-11 Syndrome Scales of the client group fulfilling all inclusion criteria (only stabilisation/psychoeducation, no confrontation, pre-post treatment measures) were high, ranging between 58.4 and 78.4%. The difference between the diagnosis via DSM-V and ICD-11 becomes apparent comparing the criteria, as 78.4% of the clients fulfilled the DSM-V criterium Intrusions whereas only 58.4% fulfilled the respective ICD-11 criterium Re-experiencing.

#### Subclinical and Non-clinical Group of Clients

The way the data aggregation was set up – providing therapy and collecting diagnostic and therapy data for all service-seeking clients without any screening for PTSD diagnosis before – gives the opportunity to explore the whole range of trauma populations in Indonesia, Cambodia and Thailand. Of the clients fulfilling all inclusion criteria, only 1.6% of the clients didn’t fulfil any DSM-V PTSD criteria at all, 44.4% of the clients were subclinical for DSM-V PTSD. For ICD-11 PTSD the prevalence is similar, only 1.6% of the clients didn’t fulfil any ICD-11 criteria, 53.2% of the clients were subclinical for ICD-11 PTSD.

### Effectiveness of Trauma Stabilisation Treatment

#### Remission Rates

This study demonstrates a treatment effect of Trauma Stabilization in significantly reducing PTSD symptoms. Trauma Stabilisation treatment was associated with very high remission rates for PTSD (91.4% for DSM V, 93.3% for ICD-11). The remission rates were also high for all PTSD criteria (ranging between 72.1 and 86.0%). Thus, a symptom reduction was achieved for all criteria, showing Trauma Stabilisation treatment was effective in reducing core PTSD symptomatology - Intrusions/Re-experiencing, Arousal/Sense of threat, Neg.Alt.Cogn&mood and Avoidance Behaviour. This study demonstrates that Trauma Stabilisation is a therapeutic agent of change on the treatment of psychological trauma including PTSD.

#### Impact of Treatment on all Subgroups

The treatment effect impacted the whole range of post-traumatic stress symptoms. Subclinical clients also improved following Trauma Stabilisation Intervention in addition to those diagnosed with PTSD and severe PTSD.

Even if subclinical and non-clinical PTSD clients were included in the analysis, results suggest Trauma Stabilisation treatment still has a high treatment effect in reducing DSM-V PTSD (Hedges’s g_av_ = 1.62) and ICD-11 PTSD (Hedges’s g_av_ = 1.39) as well as in reducing all PTSD criteria (Hedges’s g_av_ ranging between 1.08 and 1.63).

It is an advantage of the data aggregation of the Mekong I project that non-clinical and sub-clinical groups can be included in the analysis. In contrast to clients fulfilling the PTSD diagnoses pre-treatment, subclinical and non-clinical clients can get worse during the time of the treatment, fulfilling the diagnosis after treatment. This important information can’t be given in studies that screen for the diagnosis pre-treatment and only include clinical clients in the study. The rate of clients developing the diagnosis during treatment was low (1.4% of the clients developed DSM-V PTSD, 2.7% of the clients developed ICD-11 PTSD.

When looking at the tables of remission of PTSD criteria (Tables 8 and 9), it should be noted that a complete remission from all criteria is not possible for PTSD clients. There is no possibility to remise from criterion A, thus, the rate of complete remission was 0%.

#### High Effect Sizes Regardless of Diagnostic System

Regardless of the diagnoses of PTSD via DSM-IV, DSM-V or ICD-11, a very high rate of traumatized adults lost their diagnoses of PTSD after Trauma Stabilisation treatment. We can report very high effect sizes for the treatment effect on the PTSD diagnosis via DSM-V (Hedges’s g _av_ = 3.08) or ICD-11 (Hedges’s g _av_ = 3.78) as well as for the symptom reduction all criteria (DSM Hedges’s g_av_ between 2.50 and 3.09, ICD Hedges’s g_av_ between 2.88 and 3.258). They emphasize the great benefit for traumatised populations of Trauma Stabilisation Treatment. Nevertheless, the results from this study are extraordinarily high. Ferguson [44] concludes that effect size interpretation, as demonstrated by the group differences pre and post PTSD and different PTSD diagnostic criteria, indicate a strong effect (cut-off for strong effect: Hedges g = 2.70). By means of explanation to account for this one aspect could relate to the importance of the therapeutic relationship – which is also a potential agent of change. In fact the Second Task Force on Evidence-Based Therapy Relationships convened by the American Psychological Association consider this an integral factor [45]. They conclude - based on meta-analyses and reviews, that the therapy relationship accounts to the treatment success at least as much as the method/ paradigm used and thus should be explicitly addressed in practice and treatment guidelines. This recommendation is met by the Trauma Stabilisation Interventions as outlined in the ROTATE manual [34]. Norcross and Wampold [45] also describe that an adaption of the therapy relationship to specific client characteristics (in addition to diagnosis) enhanced the effectiveness of the treatment. One important aspect of adapting psychotherapy to the individual client relates to culture. Common themes regarding cultural adaptation include flexibility, adaptability, meaningfulness, empathy and traditional treatments being used alongside existing resources [46]. In their meta-analysis Smith, Rodríguez [46] show that culturally adapted mental health therapies are superior to therapies not incorporating cultural considerations. Especially Asian American clients profited from culturally adapted treatments compared to other American client groups. The Mekong Project I explicitly addressed cultural considerations in their trainings, adaptions of stabilisation techniques to fit the cultural background of the individual client were discussed and encouraged. Trauma Stabilisation Treatments have the advantage of being flexible in their use and thus, give the therapist the possibility to really adapt treatment interventions to the specific needs of their clients.

A further cultural consideration arose from the Mekong Project I therapists themselves regarding the high treatment effect sizes this research highlights. Traditionally, Cambodian, Thai and Indonesian clients don’t get any psychotherapeutic treatment at all. Thus, for all clients the therapy connected to the Mekong I Project was their first psychological treatment. With initial treatment interventions clients receive attention, validation of their trauma and posttraumatic stress problems. This is also an agent of change. Both components may contribute to a desire of the client to please the therapist. The local therapists explained that this ‘wanting to please’ also has a strong cultural component. It can’t be ruled out that at least some of the clients wanted to please their therapists and show gratitude for the subsequent treatment they received. Only further replication studies in other countries may either confirm or deny this hypothesis.

#### Putting the Effect Sizes into Context

Mekong Project I considered whether the use of a waiting list control-group was feasible, but this raised numerous ethical dilemmas and therefore was subsequently rejected. The Mekong I Project was always set up as a trauma capacity building project to meet the needs of treatment of a huge number of traumatised clients in South East Asia. Thus, the resources of the Project were used to train as many therapists as possible, in as thorough and sustainable manner as was possible. This included to offer trauma treatment to as many traumatized clients as possible – namely in the form of EMDR Therapy. This limits the interpretation of the results, as Durlak [47] emphasises effects for within-subject designs are usually much higher than for control group designs and can easily exceed 1.0. Nevertheless, Devilly and McFarlane [48] describe possibilities to still evaluate a study by comparison to existing data from wait-list controls. In their meta-analysis, the unweighted average effect size of wait-list conditions was 0.358 with a standard deviation of 0.276 and report clinical cut-offs to judge the relative efficacy of the treatment. With the effect sizes above the treatment meta-analysis mean effect size 2.42 (DSM-V Hedges’s g _av_ = 3.078, BCa 95% CI [2.875, 3.271]; ICD-11 Hedges’s g _av_ = 3.780, BCa 95% CI [3.559, 3.994]), the trauma stabilisation treatment appears to be better than best practices for PTSD treatment in the short term.

Nevertheless, the cultural context of the effect sizes isn’t taken into account in this evaluation and Devilly and McFarlane [48] describe as an alternative approach to compare the treatment group with the waiting-list group of a similar previous study. By comparing the data to an intent to treat control group aggregated in the same time and region by the same therapists with the same diagnostic tools, the waiting-list group of the RCT ROTATE [Trauma Stabilisation] study [35, 36] is a very appropriate comparator. Additionally, the cultural influences of the effect sizes are also present in this control group. The clients of this group also didn’t have any treatment at all previously and get attention and validation of their trauma for the first time. The only difference is that they get their free of charge treatments with a five-week delay. Treatment expectancy effect and an understanding of one’s own presentation due to structured assessment as well as feeling understood and supported [48], results in some reduction in the severity pf PTSD symptoms in the short term. These aspects combined with the cultural desire to please can explain the remission rate of 24.4% of DSM-V PTSD and 33.3% of ICD-11 PTSD after waiting. The effect sizes of these significant remissions are medium (DSM-V PTSD Hedges’s g _av_ = 0.594, ICD-11 PTSD Hedges’s g _av_ = 0.761).

However, the effect sizes for trauma stabilisation are almost five times the size of being on a waiting list. A direct comparison between the stabilisation treatment group and the waiting-list group via general linear model analyses reveals a very large effect for the stabilisation treatment. The explained variance between the groups is 31.03% for DSM-V PTSD and 32.38% of ICD-11 PTSD. Looking at the whole sample, regardless of the PTSD diagnosis at first measurement, also a significant difference between the clients getting worse to the second measurement becomes apparent. For the waiting-list groups five to six times more clients get worse compared to the trauma stabilisation group (DSM-V PTSD 9.1% getting worse during waiting, 1.4% getting worse during treatment, ICD-11 PTSD 14.6% getting worse during waiting, 2.7% getting worse during treatment).

The results strengthen and corroborate the earlier findings of two RCTs that effective trauma therapy can also significantly reduce posttraumatic symptoms without the need of trauma exposure [35, 36, 49]. Nevertheless, with a comparison to a waiting list group non-specific aspects of the treatment aren’t controlled. Further insights to the effectiveness of trauma stabilisation could be gained by treatment studies comparing trauma stabilisation treatment to a control group receiving treatment as usual or comparing it to trauma confrontation interventions.

### Criticism of Study

Nevertheless, the research and the results can be rightly criticized and viewed with a degree of caution. Replication studies are much needed. A compromise had to be made between conveying basic knowledge to the therapists and addressing the specific needs of the traumatised clients. With the challenge of responding to victims’ mental health needs in a post-disaster area and the priority upon mental health capacity building through training, the ability to meet methodological quality of the studies as in Western samples is quite limited [14, 50]. Thus, limitations of our study are: (a) the lack of a follow-up period (b) no external, blind rater of the PTSD-symptoms. Without a follow-up measurement, no conclusions can be drawn to the sustainability of the treatment effect. But the Mekong I Project as a capacity building project with the primary aim to meet the needs of treatment of a vast number of traumatised clients, would have been compromised by focusing at high study standards as a follow-up measurement. A comparison to the remission rate of the ROTATE RCT study where the assessments were performed by a blind to treatment allocation investigator (95.9% PTSD remission, [36]) shows that the lack of a blind rater was of little consequence.

### Advantages of the Study

#### Advantage of Local Therapists (Language, Culture)

A strength of our study is that the treatments taught in Mekong I were conducted by local therapists who had been extensively trained and supervised in Trauma Stabilisation Interventions. Consequently, Trauma Stabilisation has the benefit of being conducted using indigenous language and therefore no interpreters were necessary. Additionally, the therapists and the clients had similar cultural backgrounds, thus culture-specific interpretations of symptoms could be considered, and techniques could be culturally adapted if necessary.

#### Uniform Training of all Therapist

All therapists were trained in trauma stabilisation during the trainings of the Mekong I Project. They each had local clinical supervision which ensured treatment fidelity to the trauma stabilisation interventions used. These were deemed as being consistent with high levels of fidelity.

#### Generalizability to Real World Settings

Studies about the treatment of PTSD have been criticised for a vast number of exclusion criteria for entrance into study which decreases the generalizability of a lot of results. PTSD is associated with complex outcomes and multiple comorbid emotional, social and physical health difficulties, particularly among those who have experienced chronic traumatisation, but many published studies about Treatment Outcomes of PTSD exclude individuals with severe comorbid psychopathology [51–53]. With the data of the Mekong Project the generalizability to real world settings is extremely high, because for the clients in the Mekong Project I, there were no exclusion criteria at all. Additionally, all clients were service seekers, people that were looking for therapy. Thus, the sample of the study is very generalizable to real world settings, it consisted of clients that were looking for a trauma specific treatment.

As in real world settings, all service seeking clients received treatment and weren’t excluded if a previously screened diagnosis wasn’t completely fulfilled. With the inclusion of all service seeking clients regardless of their diagnosis, the results are not only more generalisable to real world, but also important practical implications can be drawn. The analysis of how subclinical clients develop under treatment gives important additional information about the range of the treatment effect as well as potential risks of the specific treatment.

### Trauma Stabilisation as Effective, Safe and Easy to Learn Therapy for Post Conflict Areas

Especially in areas were mental health facilities are limited and the education of therapists low, we see the need for an easy to learn and to culturally adapt treatment with no risks. Our results strengthen earlier findings that trauma therapy doesn’t necessarily require trauma exposure to be effective in reducing posttraumatic symptoms and increasing the level of functioning. Trauma Stabilisation doesn’t focus on traumatic memories directly, but resource, stabilisation and skill development. Especially in post conflict areas, with a high risk for natural disasters, this treatment can prepare the clients for future traumatic events, strengthening coping skills and enhancing resilience and potentially post-traumatic growth. A further advantage of Trauma Stabilisation Interventions is that it can be taught to paraprofessionals and allied health professionals in areas with a scarcity of mental health professionals. The results from this study are promising but more research is needed to further explore the wider impact of Trauma Stabilisation as a treatment effect in terms of clinical and economic benefits.

The study suggests that trauma stabilisation is safe, effective, efficient and sufficient in treating clinical and subclinical trauma populations with the data indicating high remission from PTSD and traumatic sequelae. Stabilisation techniques are adaptable, flexible, culturally contextualised, spiritually sensitive and individually tailored to specific needs.
